# Phenotypic and molecular cytogenetic variability in calendula (*Calendula officinalis* L.) cultivars and mutant lines obtained *via* chemical mutagenesis

**DOI:** 10.1038/s41598-019-45738-3

**Published:** 2019-06-24

**Authors:** Tatiana E. Samatadze, Svyatoslav A. Zoshchuk, Firdaus M. Hazieva, Olga Yu Yurkevich, Natalya Yu Svistunova, Alexander I. Morozov, Alexandra V. Amosova, Olga V. Muravenko

**Affiliations:** 1 Engelhardt Institute of Molecular Biology, Russian Academy of Sciences, 32 Vavilov St, Moscow, 119991 Russian Federation; 2 All-Russian Institute of Medicinal and Aromatic Plants, Federal Agency for Scientific Organizations, 7 Green St, Moscow, 117216 Russian Federation

**Keywords:** Disease prevention, Agricultural genetics

## Abstract

The morphological, meiotic and chromosomal variability were studied in two cultivars of *Calendula officinalis* L. and their mutant lines obtained though chemical mutagenesis using diethyl sulphate (DES) (0.04%, 0.08%) and dimethyl sulphate (DMS) (0.025%, 0.05%). The studied cultivars displayed different sensitivity to DMS and DES mutagens. More M1 plants with morphological changes were observed in *C*. *officinalis* cv. ‘Zolotoe more’ than in cv. ‘Rajskij sad’. DMS and DES at low concentrations had positive effects on main agro-metrical traits in both cultivars including plant height, inflorescence diameter and number of inflorescences per plant. Dose-dependent increase in number of various meiotic abnormalities was revealed in both mutant lines. Comparative karyotype analysis and FISH-based visualization of 45S and 5S rDNA indicated a high level of karyotype stability in M1 and M2 plants. Seed treatments with DMS and DES at certain concentrations resulted in higher yields of inflorescences in M1 plants compared to the control. In M2 generation, dose-dependent reduction in the yields of inflorescences was observed. Our findings demonstrate that DMS and DES at low concentrations have great potential in calendula mutation breeding.

## Introduction

A valuable medicinal plant, *Calendula officinalis* L. (*Asteraceae*), commonly known as calendula or pot marigold, is an annual aromatic herb with yellow or golden-orange flowers. This species is widely used in pharmaceutical industry, medicine, food and cosmetology^1,2^. The chemical composition of *C*. *officinalis* is well-determined. The flowers, leaves and stems contain flavonoids, xanthophylls, carotenoids, essential oils, coumarins (scopoletin) and water-soluble polysaccharides^3,4^. The raw material contains triterpenoid saponins 2–10% (oleanolic acid glycosides), triterpene alcohols (ψ-taraxasterol, taraxasterol, faradiol, arnidiol, heliantriol) and steroids. The seeds are rich in fatty acids (about 20%) including calendic acid (about 60%). It was shown that conjugated fatty acids are active substances for the treatment of obesity and also they can protect against cancer^5–7^. The flowers of *C*. *officinalis* contain rutin which has antioxidant, anti-inflammatory and anticarcinogenic activity^1,8^.

At the same time, polymorphism in active chemical compounds of the raw material was observed in *C*. *officinalis*, and also unstable active ingredients, e.g., carotinoids, were revealed^9^. Besides, calendula productivity was found to correlate with seed types and also a number of seed rows in inflorescences^10^. It is therefore important to direct research efforts towards the development of new promising *C*. *officinalis* cultivars with the desirable morpho-agronomic traits and/or biochemical profile.

Currently, for obtaining new plant cultivars with valuable economic traits, a mutagenesis approach is used alongside with transgenic crops and recombinant DNA technology. Particularly, chemical mutagenesis is an effective and simple method for obtaining valuable starting material for plant breeding as chemical mutagens (e.g., azide, diethyl sulphate, dimethyl sulphate, ethylmethane sulphonate and N-nitroso compounds) induce a high frequency of non-lethal point DNA mutations and generate novel genetic diversity in various crops^11–20^. As an example, seed treatment with low concentrations of sodium azide and diethyl sulphate was found to influence on seed germination percentage, plant height, leaf area, fresh plant weight, flowering date, inflorescence diameter and gas-exchange measurements in calendula plants of M1 and M2 generations. Also, these chemical mutagens had significant effects on total soluble proteins, acid phosphatase, and catalase activity fractions of the calendula leaves^21^.

Moreover, the quality and quantity of meiotic aberrations are considered to be reliable indicators of sub-lethal effects (doses) of chemical mutagens as well as an effective monitoring system for successful plant mutation breeding^20,22^. However, in *C*. *officinalis*, a comprehensive study of meiotic changes resulting from chemical mutagenesis has not been performed yet. Besides, currently available cytogenetic information regarding this species is rather scarce and mostly obtained by simple chromosome staining. It was shown, for example, that a karyotype of *C*. *officinalis* was composed by metacentric and submetacentric chromosomes which were small in size (1.5–3.5 µm)^23–25^. This is, probably, the reason why different karyotype formulas^23^ and chromosome numbers: 2n = 32^23–26^ and 2n = 28^27^, were described for this species. In one study, a more detailed FISH-based analysis of calendula karyotype was performed that revealed two large chromosome pairs bearing 45S rDNA loci and one pair with 5S rDNA loci^27^. However, chromosome variability in calendula mutant lines has not been investigated yet, although a comparative molecular cytogenetic analysis of *C*. *officinalis* cultivars and mutant lines could provide important information on possible karyotypic reorganization induced by chemical mutagens.

In the present study, we investigated the morphological, meiotic and chromosomal variability in two *C*. *officinalis* cultivars (cv. ‘Zolotoe more’ and cv. ‘Rajskij sad’) and their mutant lines (M1 and M2 generations) obtained though chemical mutagenesis using diethyl sulphate (DES) and dimethyl sulphate (DMS) mutagens.

## Results

### Plant morphology and productivity

**Figure 1:**
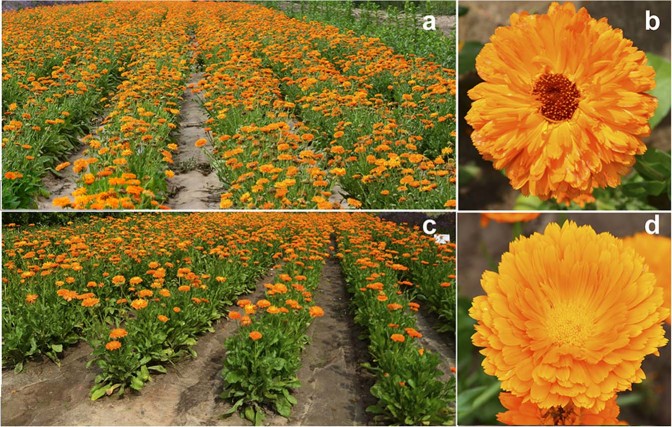
Calendula plants and inflorescences. (**a**) Plants and (**b**) inflorescences of *C*. *officinalis* cv. Zolotoe more. (**c**) Plants and (**d**) inflorescences of *C*. *officinalis* cv. Rajskij sad.

The original calendula cultivars differed in their main agro-metrical traits. Plants of cv. ‘Zolotoe more’ were considerably lower, had less inflorescence diameters and golden-yellow (with a brown center part) semi-double flowers whereas plants of cv. ‘Rajskij sad’ had larger inflorescences with bright double orange flowers (Fig. 1).

**Figure 2:**
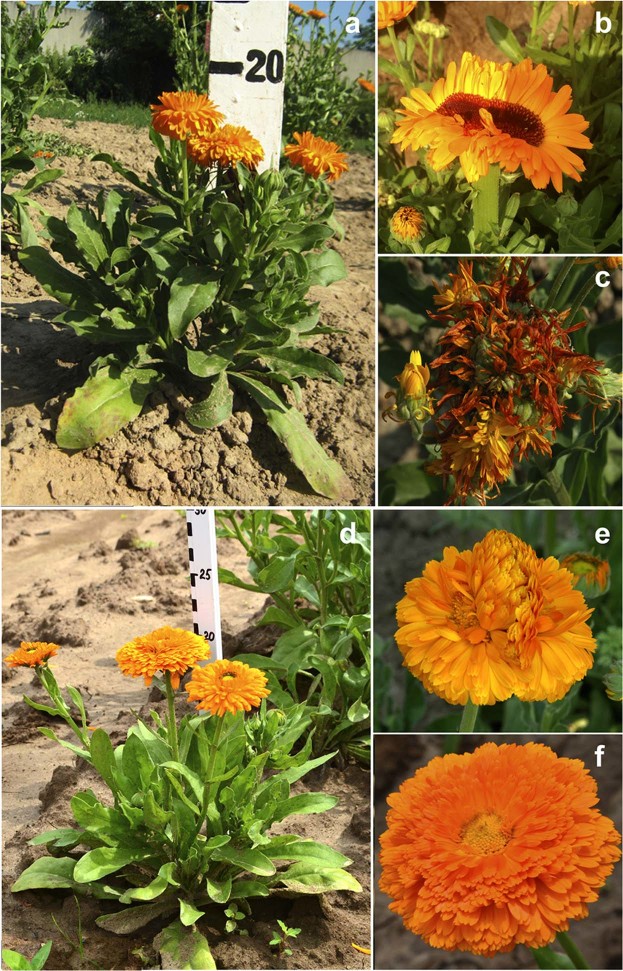
M1 dwarf plants and polymorphic inflorescences of *C*. *officinalis*. (**a**) M1 dwarf plant and (**b**,**c**) polymorphic inflorescences of *C*. *officinalis* cv. Zolotoe more. (**d**) M1 dwarf plant and (**e**,**f**) polymorphic inflorescences of *C*. *officinalis* cv. Rajskij sad.

In both studied *C*. *officinalis* cultivars, seed treatments with DMS and DES induced a number of morphological changes including plant dwarf, presence of several anthodiums in one inflorescence, polymorphism and fasciation of inflorescences, polypetalous flowers with an increased inflorescence diameter, etc. (Fig. 2). The studied calendula cultivars displayed different sensitivity to DMS and DES mutagens. More (in percentage terms) M1 plants with morphological changes were observed in *C*. *officinalis* cv. ‘Zolotoe more’ than in cv. ‘Rajskij sad’. (Table 1).

**Table 1:** Mean performance levels of vegetative parameters in M1 plants of *C*. *officinalis* cv. ‘Zolotoe more’ and cv. ‘Rajskij sad’ following exposure to DMS and DES mutagens.

Type of the experiment	Number of plants with morphological changes, %	Plant height, cm	Number of inflorescences per plant	Inflorescence diameter, cm
**cv. ‘Zolotoe more’**				
Control	—	55.2 ± 0.90	11.0 ± 0.31	5.03 ± 0.17
DMS (0.04%)	12.2 ± 1.03	56.2 ± 1.07	14.8* ± 0.51	5.73* ± 0.18
DMS (0.08%)	17.1* ± 1.56	47.8* ± 1.98	10.2 ± 0.47	5.22 ± 0.19
DES (0.25%)	21.9* ± 2.05	48.8* ± 2.02	15.0* ± 0.88	5.08 ± 0.15
DES (0.05%)	25.9* ± 2.11	50.4* ± 3.04	14.6* ± 0.48	5.34* ± 0.17
**cv. ‘Rajskij sad’**				
Control	—	57.1 ± 1.23	11.3 ± 0.36	5.84 ± 0.19
DMS (0.04%)	4.6 ± 0.33	55.5 ± 1.60	16.3* ± 0.94	6.18 ± 0.17
DMS (0.08%)	5.3 ± 0.57	57.9 ± 1.83	12.6 ± 0.41	6.35* ± 0.18
DES (0.025%)	6.2* ± 0.58	57.6 ± 1.55	14.6* ± 0.51	6.07 ± 0.22
DES (0.05%)	8.3* ± 0.78	62.1 ± 2.04	17.5* ± 1.01	6.25* ± 0.20

*Values are significantly different at P ≤ 0.05.

**Figure 3:**
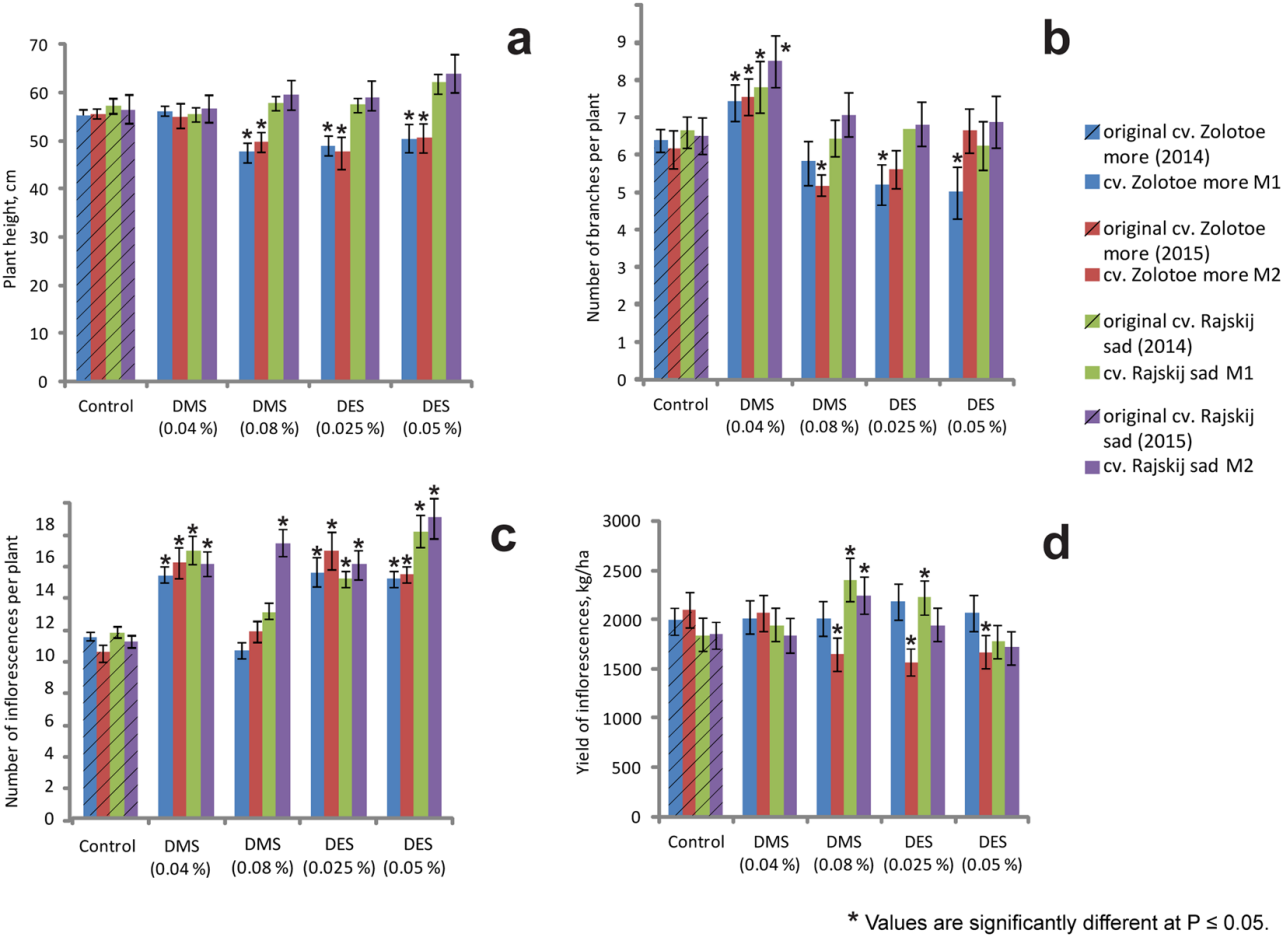
Agro-morphological traits in M1 and M2 generations of *C*. *officinalis* cv. ‘Zolotoe more’ and cv. ‘Rajskij sad’. Plant height (**a**) number of branches (**b**) and inflorescences (**c**) per plant and also yields of inflorescences (**d**) (the vertical axis) in the original cultivars (control) and also in M1 and M2 plants following exposure to DMS and DES mutagens at different concentrations (the horizontal axis).

Differences in the mean value of plant height were revealed between calendula mutant lines (Fig. 3a). In M1 and M2 plants of *C*. *officinalis* cv. ‘Zolotoe more’, plant height significantly reduced after the treatment with 0.08% DMS, 0.025% DES and 0.05% DES. In both M1 and M2 generations of cv. ‘Rajskij sad’, plant height slightly increased after the treatment with 0.08% DMS, 0.025% DES and 0.05% DES.

These chemical mutagens had no significant effects on number of branches per plant in both calendula cultivars. In M1 and M2 plants of both cultivars, the number of branches increased after the treatment with after 0.04% DMS (Fig. 3b).

DMS and DES mutagens had stimulated effects on the number of inflorescences per plant in most M1 and M2 plants of both cultivars with the exception of M1 and M2 plants of cv. ‘Zolotoe more’ after 0.08% DMS treatment (Fig. 3c). Also, treatments with DMS and DES mutagens resulted in an increase (in different degree) the inflorescences number and inflorescence diameter compared to the control (Table 1 and Fig. 3c). DMS was more effective than DES in increasing the inflorescence diameters, number of inflorescences and polypetalous flowers.

The yields of inflorescences were found to be different between the control calendula cultivars. In *C*. *officinalis* cv. ‘Zolotoe more’, it was higher (2001 kg/ha in 2014 and 2100 kg/ha in 2015) compared to cv. ‘Rajskij sad’ (1841 kg/ha in 2014 and 1853 kg/ha in 2015). The effects of different concentrations of both mutagens on calendula productivity were also different. In cv. ‘Zolotoe more’, the treatments with DMS and DES did not result in a higher yield of inflorescences in M1 plants compared to the control. In M2 generation, some reduction in the yields was observed. In cv. ‘Rajskij sad’, the treatments with 0.08% DMS (in both M1 and M2 plants) and 0.025% DES (in M1 plants) resulted in a significant increase in the yields (Fig. 3d).

### Meiosis

**Figure 4:**
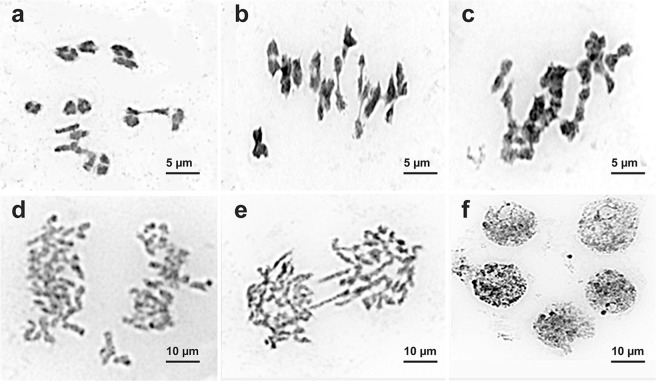
Chromosome behaviour during meiosis observed in the studied *C*. *officinalis* specimens. (**a**) diakinesis, 16^II^. (**b**) M-I, non-uniform chromosome distribution within the cell. (**c**) M-I, 9^II^ + 1^III^ + 1^IV^. (**d**) A-I, non-uniform chromosome distribution within the cell. (**e**) A-II, chromosomal bridge; (**f**) pentad.

In all studied *C*. *officinalis* specimens, analysis of meiosis indicated mostly sixteen bivalents (16^II^) at diakinesis and metaphase I (Fig. 4a). In plants of both original cultivars, the percentage of irregularities in pollen mother cells was negligible (1.13–1.28%).

**Figure 5:**
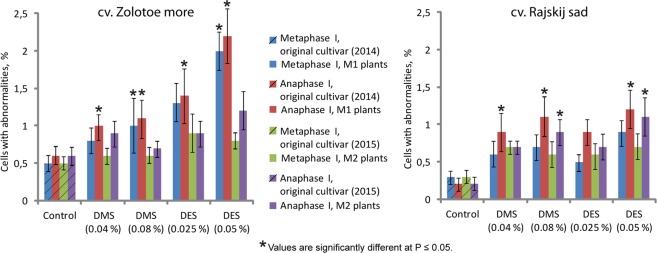
Meiotic disorders in the studied *C*. *officinalis* specimens. The percentage (the vertical axis) of the meiotic abnormalities observed in plants of the original cultivars (control) and also M1 and M2 plants following exposure to DMS and DES mutagens at different concentrations (the horizontal axis).

In calendula mutant plants, the percentage of the observed meiotic disorders was higher (2.83% for cv. ‘Zolotoe more’ and 2.13% for cv. ‘Rajskij sad’). In particular, chromosome associations (trivalents, quadrivalents and also univalents located outside the metaphase plate) were detected (Fig. 4b,c). At anaphase I and II, most cells of the studied specimens had normal chromosome disjunction (16:16) but cells with abnormalities (e.g., lagging, chaotic disjunction, bridges, chromosome fragments, etc.) were also revealed (Fig. 4d,e). At the stage of the tetrad formation, the observed irregularities included pentads, hexads and presence of 1–2 micronuclei in one of the tetrad microspores (Fig. 4f). Moreover, in M1 plants of both studied mutant lines, a dose-dependent increase in the number of meiotic abnormalities was revealed (Fig. 5).

### Karyotype structure and DAPI-banding

**Figure 6:**
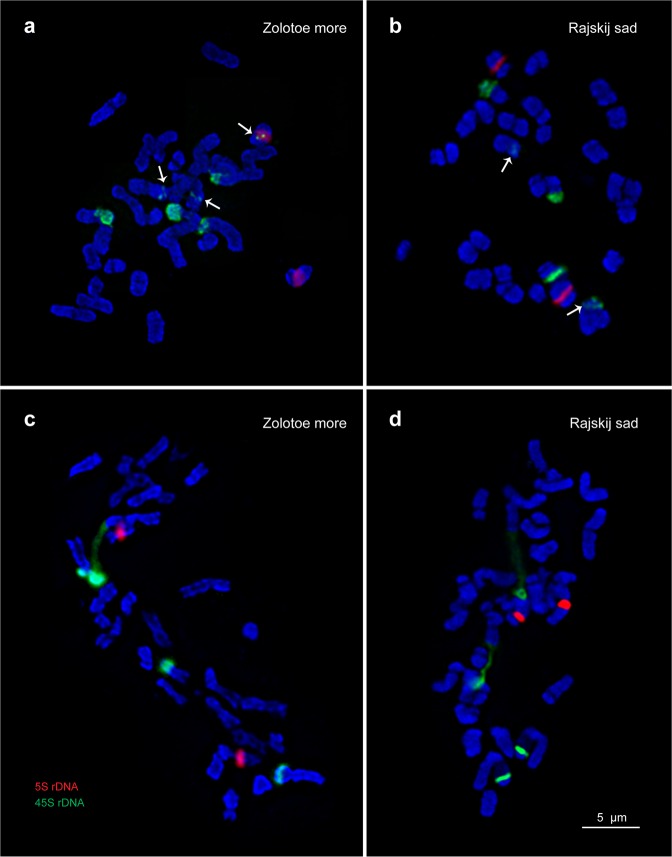
FISH-based localization of 45S and 5S rDNA on *C*. *officinalis* chromosomes. Metaphase spreads of original (control) plants of *C*. *officinalis* cv. Zolotoe more (**a**) and cv. Rajskij sad (**b**) M2 mutant plants of *C*. *officinalis* cv. Zolotoe more (**c**) and cv. Rajskij sad. (**d**) Arrows point to minor 45S rDNA loci. The correspondent probes and their pseudo-colours are specified in the lower left-hand corner. Bar − 5 μm.

**Figure 7:**
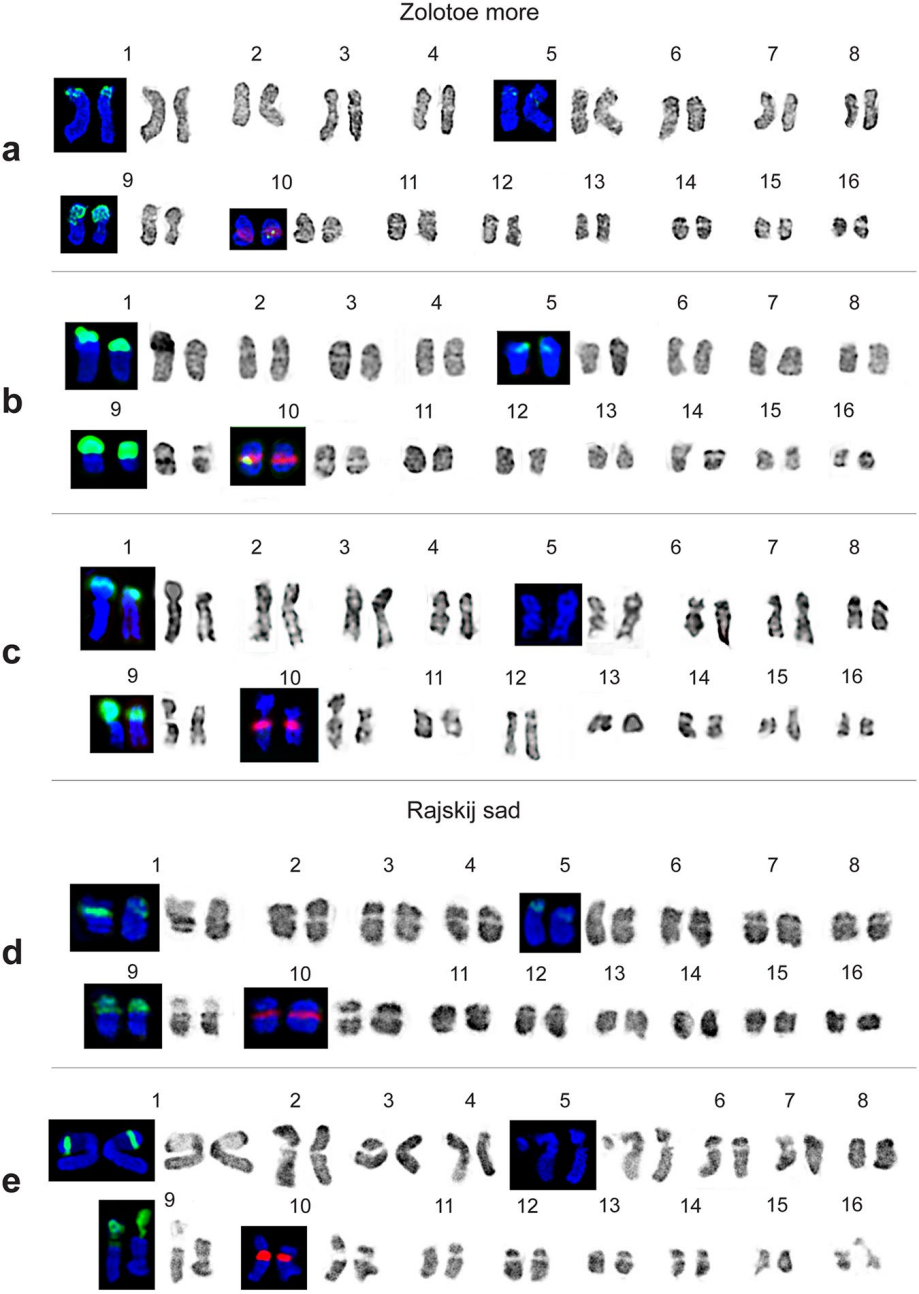
Karyotypes of *C*. *officinalis* plants. Karyograms of *C*. *officinalis* chromosomes after DAPI-banding (inverted image) and FISH (only chromosomes with hybridizations sites). (**a**) Original (control) plants of cv. Zolotoe more. (**b**) M2 plants of cv. Zolotoe more after treatment with DES 0.025% and (**c**) DMS 0.04%; (**d**) original (control) plants of cv. Rajskij sad. (**e**) M2 plants of cv. Rajskij sad after treatment with DMS 0.08%.

Karyotypes of the studied *C*. *officinalis* cultivars and mutant lines contained 2n = 32 metacentric and submetacentric chromosomes including two satellite chromosomes pairs (1 and 9) with karyotype formula K = 2(4 m + 10 sm + 2 sm^st^). The chromosome sizes ranged from 3.5 to 5.0 µm (Figs 6 and 7). Both satellite chromosomes had similar morphology with the prominent secondary constriction located in the pericentromeric region of the short arm. Satellite chromosome 1 was considerably larger than the other chromosomes in the karyotype. Also, heteromorphism of the satellite chromosomes was detected (more often, chromosome 1 in cv. ‘Zolotoe more’).

Analysis of DAPI-banding patterns in karyotypes of the studied specimens showed that large DAPI-bands were located in the pericentromeric regions of chromosomes while small and middle-sized bands were detected in the intercalary and telomeric chromosome regions (Figs 6 and 7).

### FISH

**Figure 8:**
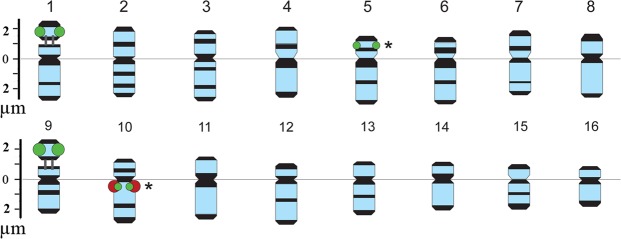
Idiograms of *C*. *officinalis* chromosomes. Idiograms of *C*. *officinalis* chromosomes showing relative sizes and positions of DAPI-bands (black segments), 45S (green) and 5S (red) rDNA sites. Asterisks indicate minor 45S rDNA loci.

In karyotypes of the studied calendula specimens, major 45S rDNA sites were localized in the short arms of two chromosome pairs 1 and 9 (Figs 6, 7 and 8). The 45S rDNA site revealed on chromosome 9 was larger (more intensive) than that observed on chromosome 1. Also, a large (bright) 5S rDNA locus was detected in the pericentromeric region of the long arm of chromosome pair 10.

Moreover, in both cultivars, a minor polymorphic 45S rDNA site was revealed in the median region of the short arm of chromosome 5. Besides, in karyotypes of cv. ‘Zolotoe more’, another minor 45S rDNA locus, colocalized with 5S rDNA site, was revealed in one homolog of chromosome 10. In karyotypes of cv. ‘Rajskij sad’, this minor 45S rDNA locus was not detected (Figs 6, 7 and 8).

Based on chromosome morphology, DAPI-banding patterns and distribution of 45S and 5S rDNA sites, all chromosome pairs in the karyotypes were identified (for the first time) and chromosome idiograms of *C*. *officinalis* were constructed (Fig. 8).

In M2 plants of cv. ‘Zolotoe more’, the minor 45S rDNA sites observed on chromosomes 5 and 10 were polymorphic. After the treatment with 0.025% DES and 0.04% DMS, the pattern of chromosomal distribution of 45S and 5S rDNA sites was similar to that revealed in the control plants (cv. ‘Zolotoe more’). At higher mutagen concentrations (e.g., 0.08% DMS), the minor 45S rDNA sites were not visualized. In M2 plants of cv. ‘Rajskij sad’, the patterns of chromosomal distribution of 45S and 5S rDNA loci were mostly similar to the control plants. The exception was the treatment with 0.04% DMS. In this case, the minor 45S rDNA locus was not visualized.

In the studied *C*. *officinalis* karyotypes, any chromosomal rearrangements were not revealed.

## Discussion

In the present study, DMS and DES mutagens stimulated the diversity in morphological characters of *C*. *officinalis* including plant height, number of inflorescences, inflorescence diameter, etc. Currently, an optimal plant height is a desirable agronomic trait that contributes to a high yield. Besides, the variation in calendula plant height is one of the initial associated traits in response to a mutagen treatment even at low concentrations^21,28^. In this study, DMS and DES treatments resulted in plant height changes in both *C*. *officinalis* cultivars, and our results are consistent with previous findings on other flowering plants including *Salvia splendens*^29^, *Euonymus japonicus*^30^ and *Amaranthus*^31^. At the same time, the observed variations in this important agronomic trait were dose-dependent, and those changes were different between the examined calendula cultivars. Considered all, the mean value of the plant height was less in M1 and M2 plants of cv. ‘Zolotoe more’ compared to the original cultivar (control). However, in M1 and M2 plants of cv. ‘Rajskij sad’, this parameter was more than in the control. These differences are probably related to different levels of plasticity in plants of the studied cultivars, and in some cases, mutagen-induced damages of the plant cells could be repaired at the initial ontogenesis stages^10^. In general, both examined mutagens induced the increase (in different degrees) in number of inflorescences, inflorescence diameter, shoots, and doubleness in flowers, and these findings agreed with the earlier reported data on DMS and DES mutagenic effects^10,32^. It is important to note, however, that the weather environment factors during growing period should also be considered in making an assessment of these mutagenic effects^21,33^.

Many chemical mutagens were shown to influence the plant genome and cause the meiotic disorders manifested themselves as typical chromosome aberrations (chromosome fragments, bridges, lagging, etc.) as well as mass fragmentation, nondisjunction, chromosome stickiness and other abnormalities^20,34^. Deviations from the normal bivalent conjugation could be displayed as univalent and multivalent formation at metaphase I stage^35,36^. In the studied calendula lines, alongside with normal meiotic segregation patterns, we observed various chromosomal associations at metaphase I (trivalents, quadrivalents and also univalents located aside of the plate or near the poles of the microsporocyte). The univalent formation induced by mutagens was supposed to be a result of changes in the structure of chromosomes followed by the reduction of chiasma frequency due to restriction of pairing to homologs^37^. The appearance of the multivalent associations is considered to be caused by mutagen-induced chromosome breaks followed by the reciprocal translocation joining^38^. Alternatively, these associations can be a result of chromosome mismatches and breakages which lead to translocations and inversions^37,39–43^.

Chromosome nondisjunction, occurred at anaphase I, is considered to be a serious meiotic abnormality which resulted in chromosome loss as well as unequal distribution of genetic material. Most cells of the studied here specimens had normal chromosome disjunction (16:16) at anaphase I and anaphase II, however, cells with abnormalities (e.g., chromosome lagging, chaotic disjunction, bridges, fragments, etc.) were also revealed. These disorders could be related to the paracentric inversions as previously described in tomatoes and *Nigella sativa*^44^.

Thus, in the present study, the analysis of the chromosome behaviour during meiosis revealed an increased number of microsporocytes with various meiotic disorders in calendula mutant lines if compared with the control. Moreover, a high level of phenotypic variability observed in the examined mutant lines could also be related to mutagen-induced chromosome mutations. An estimation of cytological abnormalities and their magnitude during mitosis or meiosis is believed to be a key component to determine the effect of a mutagen and also species sensitivity to sublethal doses in mutation breeding experiments^20^. At the same time, zygotes with chromosome mutations (appeared due to meiosis abnormalities) were shown not always to produce viable seeds^45^. Considering that karyotypes in M2 plants did not differ in chromosome number and morphology from the original calendula plants, our findings indicates that DMS and DES at low concentrations are rather effective mutagens for calendula breeding, which had positive effects on vegetative parameters without dramatic consequences for the calendula genome.

Basically, the results of karyotype study in the calendula cultivars agreed with earlier reported data which were mainly based on simple monochrome staining. At the same time, the application of DNA intercalator 9-AMA allowed us to obtain longer by half chromosomes in metaphase plates and also specify the morphology of *C*. *officinalis* chromosomes. As a result, the karyotype formula of *C*. *officinalis* reported here differed from that described earlier^23^ by a ratio of metacentric and submetacentric chromosomes. Moreover, we have identified two satellite chromosomes in the karyotypes and also specified the chromosome number of *C*. *officinalis* as both numbers 2n = 32^23–26^ and 2n = 28^27^ have been described before.

DAPI-banding was shown to reveal AT-rich heterochromatic regions^46–48^. In plant species with small chromosomes, chromosome banding patterns are usually rather poor which makes it difficult to identify precisely each chromosome in a karyotype^49,50^. Typically, in small-sized chromosomes, large heterochromatic bands located in the pericentromeric regions, small telomeric bands and few small intercalary bands are revealed^51–54^. The application of DNA intercalator 9-AMA allowed us to increase the resolution of chromosome DAPI-banding patterns, improve accuracy of physical mapping of rRNA genes, and then use these markers for analysis of calendula chromosomes. As a result, all chromosomes in karyotypes of *C*. *officinalis* original cultivars and mutant lines were identified for the first time. The results of FISH-based analysis of chromosomal distribution of major 45S and 5S rDNA in the original *C*. *officinalis* cultivars agreed with the currently available data^27^. Moreover, we detected minor 45S rDNA loci on chromosome 5 (both cultivars) and on one homolog of chromosome 10 (cv. ‘Zolotoe more’). These additional 45S rDNA loci were probably related to intraspecific genomic polymorphism.

In M2 plants, the analysis of chromosomal distribution of 45S and 5S rDNA sites revealed dose-dependent differences in localization of these minor 45S rDNA sites on chromosomes 5 and 10. Moreover, the plants of cv. ‘Zolotoe more’ were more sensitive to these mutagenic effects than cv. ‘Rajskij sad’.

The optimal conditions for pre-sowing seed treatments with chemical mutagens was shown to result in improved vegetative growth and flowering characteristics, increased yields and stress tolerance in comparison to normal plants^19,55^. Both DMS and DES mutagens were successfully applied for improving the vital characters of the floricultural crops including calendula^10,21^. Our results show that DMS and DES had positive effects on valuable agro-morphological traits in *C*. *officinalis*, and therefore these mutagens have great potential for improvements in the viability, yield and quality of calendula. Particularly, seed treatments with 0.08% DMS and 0.025% DES resulted in higher yields of inflorescences in M1 and M2 plants of cv. ‘Rajskij sad’ compared to the control. In cv. ‘Zolotoe more’, slight increase in the yields of inflorescences in M1 plants and dose-dependent reduction of this parameter in M2 plants were observed. Therefore, these chemical mutagens can stimulate useful traits in calendula genotypes, and this is very important for developing new promising cultivars in order to meet market demands. Currently, induced mutagenesis has become widespread in plant mutation breeding to solve a very wide range of problems, and particularly, this method is successfully used for developing improved crops within the *Asteraceae* family including *Dendranthema grandiflora* Tzvelev, *Artemisia pallens* Bess. and *Helianthus annuus* L.^56–59^.

Thus, our findings demonstrated that DMS and DES at certain concentrations had positive effects on morphological and yield attributes of calendula which could be useful for further breeding. At the same time, the studied *C*. *officinalis* cultivars displayed a different sensitivity to DMS and DES mutagens. This could be indicative of genetic polymorphism among *C*. *officinalis* cultivars, which correlates with variability in the cultivar characteristics. Therefore, a careful testing of these mutagens at different concentrations is required for successive realization of plant breeding programs.

## Methods

### Plant material

The calendula plants were grown in the Botanical garden of the All-Russian Institute of medicinal and aromatic plants (Moscow, Russian Federation) during two successive experimental seasons (summer 2014 and summer 2015). At late April 2014, seeds of *C*. *officinalis* cv. ‘Zolotoe more’ (K-36829, Russian Federation) and cv. ‘Rajskij sad’ (K-36837, Russian Federation) were treated with aqueous solutions of DMS (0.04% and 0.08%) and DES (0.025% and 0.05%) for 18 h. The control seeds were treated with water. The types of mutagens, their concentrations and treatment time were optimized based on our previous studies^10^ as well as early described data on application of chemical mutagens in breeding of horticultural crops^10,21,32^. After the mutagen treatment, the seeds were washed in water and sowed into the greenhouse for obtaining seedlings (M1 generation). At the stage of 4–5 true leaves, the seedlings were planted in the field in crop geometry of 60 cm × 30 cm according to the earlier described approach^60^. Plant vegetative parameters were measured at the stage of flower maturity of the plants. Seeds from the individual M1 plants were harvested and then used both for cytogenetic assays and for cultivation of M2 plants in the next growing season. At early May 2015, these seeds were sowed into the greenhouse and then grown in the field in the same way as M1 plants. The productivity (yield of inflorescences) of calendula plants was determined as a total sum of ten-round harvesting of inflorescences during the flowering period according to the earlier described approach^60^. Statistical data analysis was performed using standard functions of Microsoft Excel 2013.

### Chromosome slide preparation

For FISH assays, the modified technique of chromosome spread preparation from root tips was applied. The seeds were germinated in Petri dishes on the moist filter paper at room temperature. Root tips (of 0.5–1 cm) were excised and stored for 16–20 h in ice-cold water with 1 µg/mL of 9-aminoacridine (9-AMA) (Sigma, St. Louis, USA) to inhibit chromosome condensation process and accumulate prometaphase chromosomes^51,61,62^. After the pre-treatment, the root tips were fixed in ethanol:acetic acid (3:1) fixative for 48 h at room temperature. Before squashing, the roots were transferred into 1% acetocarmine solution in 45% acetic acid for 15 min. The cover slips were removed after freezing in liquid nitrogen. The slides were dehydrated in 96% ethanol and stored at −20 °C until use.

For meiotic chromosome preparation, young floral buds (prefoliation) were fixed in ethanol:acetic acid (3:1) fixative for 30 min at 4 °C and then chromosome spreads were prepared as previously described^63^. After freezing in liquid nitrogen, the cover glasses were removed, and the slides were stored in 96% ethanol at −20 °C until use.

### DNA probe preparation and FISH procedure

The following probes were used for FISH:

1. pTa71 - a 9-kb-long sequence of common wheat encoding 18S, 5.8S, and 26S rRNA genes including spacers^64^. This DNA probe was labelled directly with SpectrumAqua (Abbott Molecular, Wiesbaden, Germany).
2. pTa794 - a 420-bp-long sequence of wheat containing the 5S rRNA gene and intergenic spacer^65^. This DNA probe was labelled directly with SpectrumRed fluorochrome (Abbott Molecular, Wiesbaden, Germany).

FISH procedure was carried out according to Muravenko *et al*.^51^. After overnight hybridization, the slides were washed as described previously^66^.

### DAPI-banding

After FISH procedure, chromosome slides were stained with 0.1 μg/ml DAPI (4′,6-diamidino-2-phenylindole) (Serva, Heidelberg, Germany) dissolved in Vectashield medium (Vector laboratories, Peterborough, UK).

### Chromosome analysis

The slides were examined using an Olympus BX-61 epifluorescence microscope (Olympus, Tokyo, Japan). Images were captured with monochrome charge-coupled device camera (Cool Snap, Roper Scientific, Sarasota, USA). Then they were processed with Adobe Photoshop 10.0 software (Adobe, Birmingham, USA). At least 15 metaphase plates were investigated for each specimen. For meiosis analysis, at least 200 cells (10 plants) of each sample were analyzed. The meiotic chromosome preparations were analyzed as described previously^52^.

## Data Availability

All data generated or analyzed during this study are included in this published article.
